# Congenital thrombotic thrombocytopenic purpura (TTP) with placental abruption despite maternal improvement: a case report

**DOI:** 10.1186/s12884-020-03051-2

**Published:** 2020-06-15

**Authors:** Marti D. Soffer, Pavan K. Bendapudi, Drucilla J. Roberts, P. Kaitlyn Edelson, David J. Kuter, Jeffrey L. Ecker, Allison Bryant, Ilona T. Goldfarb

**Affiliations:** 1 Department of Obstetrics, Gynecology, and Reproductive Biology, Division of Maternal Fetal Medicine, The Massachusetts General Hospital, Boston, MA USA; 2 Department of Hematology/Oncology, The Massachusetts General Hospital, Boston, MA USA; 3 Department of Pathology, The Massachusetts General Hospital, Boston, MA USA

**Keywords:** TTP pregnancy, Placental abruption, Antenatal surveillance, Case report

## Abstract

**Background:**

Thrombotic thrombocytopenic purpura (TTP) is a rare but serious complication in pregnancy that places the mother and fetus at high risk for morbidity and mortality. This case illustrates novel pregnancy complications associated with this rare medical condition.

**Case presentation:**

A 31-year-old G3P0020 at 28 weeks and 1 day was admitted with severe thrombocytopenia and was ultimately diagnosed with TTP. With therapeutic plasma exchange (TPE), maternal status improved. At 28 weeks 6 days, however, non-reassuring fetal testing prompted cesarean delivery with placental abruption noted intraoperatively. Pathology examination confirmed placental abruption and also revealed multiple placental infarcts.

**Conclusion:**

While medical management of TTP can significantly improve the health of the mother, this case highlights the potential role of TTP in abruption and other placental pathology and thus, the need for close fetal surveillance throughout an affected pregnancy.

## Background

Thrombocytopenia in pregnancy is common, usually mild, and most often, attributed to gestational thrombocytopenia which can be monitored without treatment or adverse outcome. On rarer occasions, thrombocytopenia is the first indicator of a more severe disease process with potential for both fetal and maternal morbidity or even mortality.

Thrombotic thrombocytopenic purpura (TTP) is a rare hematologic condition that leads to severe thrombocytopenia and occlusive microangiopathywith potential sequalae including neurological abnormalities, renal insufficiency, fevers, thrombocytopenia, end organ damage and death if untreated. The disease may be either congenital or acquired, and is caused by decreased activity of ADAMST13, a von Willebrand factor cleaving protease [1]. ADAMST13 activity may be decreased by the presence of an inhibitor or autoantibody as in the case of acquired TTP or, in the case of hereditary TTP, as the result of decreased production from a genetic mutation, a circumstance also known as Upshaw Shulman Syndrome (USS). Treatment involves therapeutic plasma exchange (TPE) together with immune suppression in autoimmune (acquired) TTP or replacement of deficient ADAMTS13 with fresh frozen plasma (FFP) infusions in the case of USS [2]. Pregnancy has been known to perpetuate both acquired and congenital disease processes, and result in both significant maternal and fetal complications [3, 4].

Prior case reports have focused primarily on maternal complications with only a few reports of fetal outcomes. When poor fetal outcomes are reported they have been associated with severe maternal disease [3–5]. We present a case of maternal congenital TTP diagnosed during pregnancy in the early third trimester with appropriate response to TPE but deterioration of fetal well-being requiring preterm delivery.

## Case presentation

A 31-year-old G3P0020 Caucasian woman at 28 weeks and 1 day was found to have severe thrombocytopenia on routine third trimester laboratory testing. She reported easy bruising over the previous week but denied additional symptoms including headache, blurry vision, epigastric pain. Pregnancy had been uncomplicated until this point with no contributory medical, surgical, or family history and she was transferred to our facility for further care. She was normotensive on presentation and laboratory evaluation revealed a hematocrit (Hct) of 24.9%, platelets (plt) of 19 × 10^9^/L, total bilirubin of 1.8 mg/dL, AST 33 U/L, ALT 20 U/L, creatinine 1.05 mg/dL, LDH 628 U/L, normal PT/PTT. Peripheral smear showed schistocytes and an ADAMST13 level was drawn. Obstetrical ultrasound revealed a singleton fetus in the breech presentation with adequate fluid and an estimated fetal weight of 850 g (16th percentile for the gestational age). Continuous electronic fetal monitoring on admission showed intermittent late decelerations every 1–2 h with recovery. The patient did not experience vaginal bleeding, uterine contractions, or leakage of fluid during initial evaluation or throughout her hospital course.

The Hematology and Transfusion Medicine services were consulted and they believed the leading diagnosis to be TTP. TPE was initiated empirically and transfusions of FFP and packed red blood cells (pRBC) were given due to her severe anemia. The patient was also started on a course of betamethasone for fetal lung maturity. Maternal vitals and the fetal heart rate were monitored continuously throughout the TPE given concern for change in fetal status with fluid shifts. No fetal concerns were noted during TPE.

Over the next several days, lab evaluations, repeat TPE through a central catheter placed by interventional radiology, and fetal assessments continued throughout the treatment course. The patient remained normotensive and intermittently tachycardic; platelet counts showed clinical response to the TPE. She was started on a course of prednisone 70 mg/day to further treat the TTP and improve her platelet counts. The fetus had reactive and overall reassuring nonstress testing throughout the subsequent days of treatment as platelet counts began to improve (see Table 1). On hospital day 4, testing for ADAMST13 activity returned and was < 5% and testing was negative for an inhibitor, consistent with a diagnosis of hereditary TTP. Confirmatory genetic testing was sent and a prednisone taper was initiated.

**Table 1:** Laboratory Trends Prior to Delivery

	Outside Hospital labs	Hospital Day 1	Treatment Day 1	Day 2	Day 3	Day 4	Day 5
Hct	25	23	25	25	25	288	31.9
Plt	19	16	38	59	126	161	168
LDH		628	604	317	217	293	291

On hospital day 5, at 28 weeks and 6 days, the fetal nonstress test demonstrated intermittent late and variable decelerations throughout TPE. Following treatment, fetal tachycardia was noted to the 170’s and the tracing evolved to demonstrate minimal variability. Bedside ultrasound showed a fetus in breech presentation with normal amniotic fluid volume. No gross fetal movements or evidence of muscle tone were noted, however. Given the ultrasound and NST findings, the decision was made to proceed with delivery via primary cesarean section. Platelet count was 168,000/μL with normal coagulation studies, and a hematocrit of 31.6. The patient was started on magnesium for fetal neuroprotection prior to delivery.

The patient underwent a primary cesarean section through a Pfannensteil skin incision under spinal anesthesia. Evaluation of the uterus at the time of surgery revealed a developed lower segment which allowed for a low transverse hysterotomy. Large fresh blood clots were noted upon entry into the uterine cavity and on evaluation of the placenta, concerning for clinical abruption. Intraoperative EBL was 1000 mL. The male fetus was delivered from the breech position with Apgars assigned as 4, 6, and 8 at 1, 5 and 10 min, respectively, with a birthweight of 1085 g. Umbilical cord arterial pH was 7.02 with a base excess of 12, venous pH was 7.08. The infant was transferred to the NICU for respiratory support and prematurity. Subsequent fetal testing showed ADAMST13 activity of 35% and no evidence of an inhibitor. Neonatal hematocrit was 52.3%.

Postoperatively, the patient was monitored closely and required 7 days of daily TPE and 4 units of pRBC. She had no evidence of ongoing blood loss and continued to improve throughout her hospital course. Elevated blood pressures on postoperative day 5 though without other features of preeclampsia and blood pressures were stabilized with labetalol 200 mg BID. She was discharged on postoperative day 8 with a stable hematocrit of 31.5 and platelets of 317 × 10^9^/L and plans for close follow up with both hematology and obstetrics.

As noted above, maternal evaluation for the underlying cause of TTP included genetic testing which revealed a compound heterozygous state with mutations in exons 24 (g.136319670C > T) and 13 (g.136302889_13602890del) of the ADAMTS13 locus. In particular, this patient’s exon 24 mutation is commonly associated with congenital TTP often unmasked for the first time during pregnancy [6]. These results confirm the presence of congenital TTP (USS) in this patient.

**Fig. 1:**
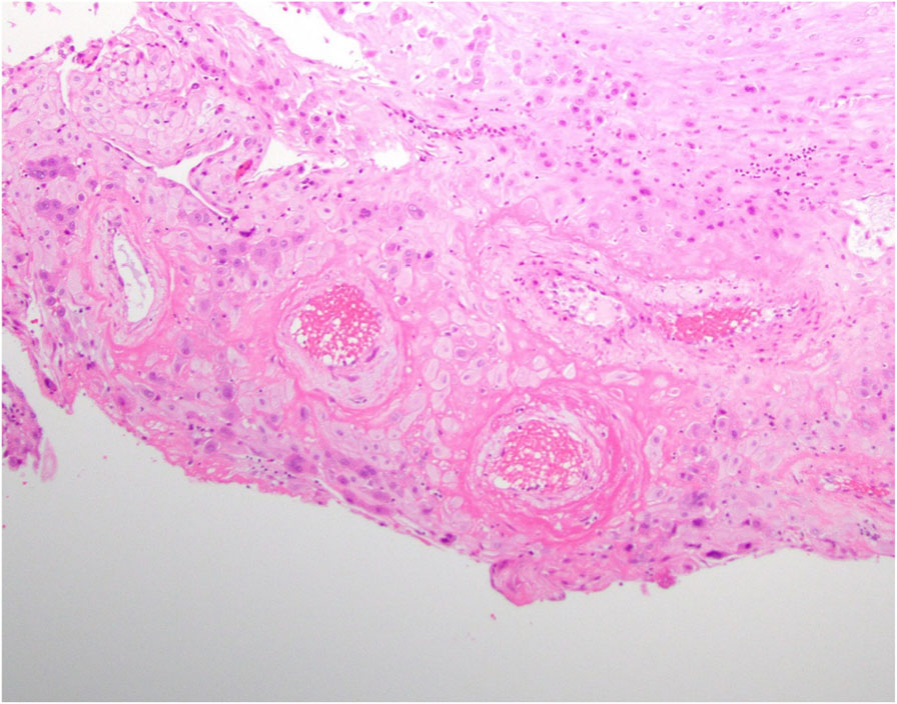
decidual arteriopathy with acute atherosis

**Fig. 2:**
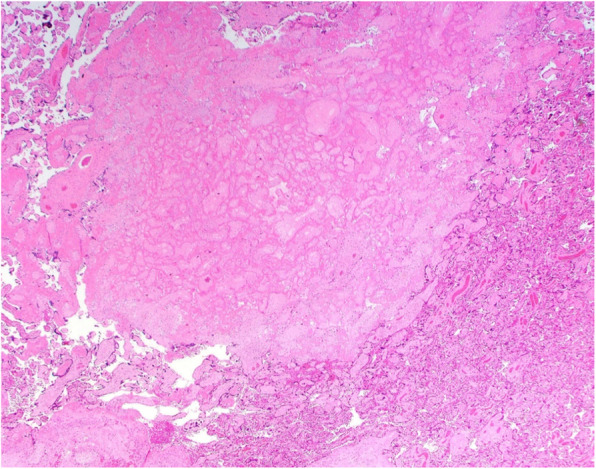
multiple placental infarcts with usual and hypertensive types.

Placental pathology was read by a placental pathology expert (DJR) and showed a disrupted mature placenta weighing 150 g (< 10 percentile for gestational age if the placenta was complete). A peripheral cord insertion and accessory lobe with a patent membranous vessel were noted. There was evidence of maternal vascular malperfusion in the small placental weight, decidual arteriopathy with acute atherosis (Fig. 1), accelerated villous maturation, multiple placental infarcts with both usual and hypertensive types (Fig. 2) occupying 15% of the placenta. Gross features of acute abruption were also noted. These findings were consistent with placental abruption, and maternal vascular compromise to the fetus.

As an outpatient, the patient continued to receive intermittent plasma infusions and progressed well with evidence of recovery from her rapid disease process as demonstrated by normalized platelet count, no continued bruising or evidence of blood loss, and normal coagulation studies. Her postoperative hypertension was managed as an outpatient by her primary OB provider.

## Discussion and conclusions

We present this case to discuss a rare disease entity and the potential role for obstetrical monitoring and surveillance to improve fetal outcomes. Thrombotic thrombocytopenic purpura in pregnancy is a rare but potentially fatal condition for both the patient and her fetus if left untreated. The disease is a known mimicker of preeclampsia, and often the two entities can coexist within the same patient thus requiring a low index of suspicion to initiate further workup [7]. TTP, whether congenital or acquired, is often triggered by pregnancy [3–5]. Swift recognition of TTP is needed to ensure prompt treatment with TPE. In our patient, the diagnosis of congenital TTP (USS) was made and ultimately confirmed on genetic testing of her ADAMTS13 locus. Although exceedingly rare in the general population and as a cause of TTP overall USS is a surprisingly common etiology of TTP presenting in pregnancy, responsible for 24–66% of cases [3, 4]. Therefore, clinicians should have a high index of suspicion for USS and undertake genetic testing in women presenting with a first episode of TTP during pregnancy.

Treatment with TPE drastically decreases the rates of maternal mortality otherwise resulting from TTP [7]; however data is limited regarding fetal outcomes during an index pregnancy. The available data suggests that TTP predominantly is diagnosed in the late second to early third trimester [7], and fetal survival is more likely if TTP is recognized and treated beyond 30 weeks gestation [3]. It has been posited that late presentations of TTP are associated with more mild disease and greater placental reserve, while pregnancies in the second trimester are likely to have severe placental dysfunction from the underlying disease process leading to severe fetal growth restriction (FGR) and stillbirth [3, 4]. The underlying cause of these findings is unknown, however, it suggests that earlier disease presentation allows for progression of the disease within the placenta and therefore in the growing fetus, while later presentations affect a placenta that has developed normally and is able to better withstand insult in the third trimester.

Fujimura reviewed 15 pregnancies with congenital TTP and reported 8 stillbirths or very early neonatal deaths, the majority of whom were delivered in the second to early third trimester [5]. However, the majority of patients included in this review did not receive TPE or plasma infusion therapy for their disease. In a French review of patients with acquired TTP, there was a 60% risk of stillbirth with better fetal prognosis if TTP developed and was recognized in the third trimester [4]. Older series have been reported showing improved maternal and subsequent fetal mortality with plasma therapies, but do not further characterize the patients studied by subtype of TTP [8, 9]. This distinction is important as the cornerstone of treatment for patients with congenital TTP is plasma infusion and not TPE as is required in patients with acquired TTP. However, this may not be a distinction made expeditiously in an index pregnancy given the time required to receive a genetic diagnosis.

In work by Scully et al. (2014), 3 women with congenital TTP presenting prior to 20 weeks were treated and carried to term without adverse fetal outcomes [3]. Among 17 women presenting later in gestation, 6 presented between 20 and 29 weeks with one live birth and the remaining experienced stillbirth, and 11 presented after 30 weeks and had live births though not all were treated with plasma therapy.

For pregnancies affected by known maternal congenital TTP, treatment in future pregnancies with FFP infusions leads not only to improved maternal outcomes, but also to improved fetal outcomes [3]. Patients with index pregnancies affected by acute TTP demonstrated changes such as villous hypoplasia and atherosis in those who remained untreated indicating placenta ischemia. These histologic changes were not seen in future pregnancies if treated appropriately and aggressively, and fetal outcomes were all normal [3].

As the available literature is limited, it is not known whether treatment with TPE or plasma infusions during the pregnancy will lead to improved fetal outcomes. As described, there is a high incidence of stillbirth in patients with TTP, most pronounced if the diagnosis is made in the late second trimester. Further confounding the discussion regarding fetal outcomes with TTP is the known association with comorbid preeclampsia [10], also experienced by our patient though in the postpartum period. It is known that preeclampsia is associated with FGR, placental abruption, and poor fetal outcomes, however, it is not known to what degree comorbid TTP may contribute to these outcomes. It is important to note, however, that while the disease process of preeclampsia is improved with delivery of the fetus, the same is not true for TTP. Therefore, decisions regarding delivery in the case of significant maternal disease must be made judiciously.

In a previously published case report, Datta et al. [11] describe a patient presenting at term with placental abruption necessitating urgent delivery and the patient’s postoperative course was complicated by thrombocytopenia and diagnosis of congenital TTP. Datta et al. highlighted the importance of maintaining a high index of suspicion for TTP with maternal decompensation and unexplained thrombocytopenia. The current work adds to the possible link between the underlying disorder and the development of placental abruption by describing the development of abruption during the disease flare and even during a period of maternal stabilization. Both cases highlight the presence of placental abruptions in the absence of other risk factors for this pathology, namely hypertension, preeclampsia, previous abruption, smoking, illicit drug use. Together with this prior case report, our case highlights the need for close monitoring of pregnancies affected by TTP for the development of adverse obstetrical outcomes.

The case presented here illustrates the need to consider fetal monitoring in pregnancies complicated by TTP, regardless of the maternal response to appropriate TPE and or plasma infusion, and irrespective of comorbid preeclampsia. Though the case presented illustrates swift diagnosis, treatment, and maternal stabilization of TTP, it also highlights the unexpected change in fetal status after maternal improvement necessitating delivery. While the optimal interval and duration of fetal monitoring are unknown, this case suggests that pregnancies affected by maternal TTP require ongoing fetal surveillance to determine the timing of delivery, potential role for antenatal steroid administration, and assure optimal neonatal outcomes.

## Data Availability

Not applicable.

## Abbreviations

TTP: Thrombotic thrombocytopenic purpura
FGR: Fetal growth restriction
FFP: Fresh frozen plasma
pRBC: Packed red blood cells
NST: Non stress test
NICU: Neonatal intensive care unit
USS: Upshaw schulman syndrome
TPE: Therapeutic plasma exchange
